# Evaluation of luteolytic activity and reproductive outcomes in ewes following single and split dose cloprostenol during the breeding season

**DOI:** 10.1007/s11250-026-04916-y

**Published:** 2026-02-12

**Authors:** Neffel Kürşat Akbulut, Yavuz Kal, Mesut Kırbaş, Fatih Aladağ, Hasan Alkan

**Affiliations:** 1 Department of Obstetrics and Gynaecology, Faculty of Veterinary Medicine, Necmettin Erbakan University, Konya, Ereğli Turkey; 2 Bahri Dağdaş International Agricultural Research Institute Karatay, Konya, Turkey; 3 Department of Obstetrics and Gynaecology, Faculty of Veterinary Medicine, Selçuk University, Konya, Turkey

**Keywords:** Prostaglandin F_2α_, Luteolytic effects, Progesterone, Reproductive parameters, Ewes

## Abstract

The objective of the present study was to investigate the luteolytic effects of different and split doses of cloprostenol administered during the mid-luteal phase of the estrous cycle in ewes. For this purpose, 147 Anatolian Merino sheep aged 3–5 years were used in the breeding season. The ewes were assigned to three groups. A pre-synchronization was performed to synchronize the estrous periods of the ewes before the treatments were started in all groups, and estrus was monitored. The day of oestrus was accepted as day 0. Afterwards, on day 12 of the estrous cycle, Group I (1 dose) was administered 250 µg of cloprostenol; Group II (½ dose) received 125 µg of cloprostenol; and Group III (2 dose ) was administered two doses of cloprostenol: 125 µg on day 12 and an additional 125 µg on day 13, with a 24-hour interval between administrations. No significant differences were observed in estrus rates or the timing of estrus onset among the different prostaglandin administration protocols in ewes. However, in the group that received prostaglandin in split doses, 95% of the ewes exhibited estrus within the first 48 h (*P* < 0.05). The highest pregnancy rate was observed in the group that received a 1 dose of prostaglandin, although there was no statistically significant difference when compared to the group receiving 2 dose. Additionally, progesterone levels did not show time-dependent variation. In conclusion, although different prostaglandin administration protocols did not affect overall estrus rates, it was determined that the majority of estrus responses occurred within the first 48 h following 2 dose prostaglandin administration. Moreover, pregnancy rates were found to be similar in the 1 dose and 2 dose groups, whereas pregnancy rates decreased following administration of a ½ dose of prostaglandin. Based on these findings, it was observed that sheep responded to all doses during the mid-luteal phase of the estrus cycle.

**Supplementary Information:**

The online version contains supplementary material available at https://doi.org/10.1007/s11250-026-04916-y.

## Introduction

Prostaglandin F_2α_ (PGF_2α_) and its synthetic analogs have been studied for many years due to their strong luteolytic effect (Fierro et al. 2013). Prostaglandins are used in ewes between days 5 and 14 of the estrous cycle for this purpose (Acritopoulou and Haresign 1980; Fierro et al. 2013). The existing literature on this subject is unclear regarding the timing of the initial response of sheep to PGF_2α_. This uncertainty is partly due to the response to exogenous PGF_2α_ depending on factors such as the day, dose, exposure frequency, and route (Pope and Cárdenas 2004). PGF_2α_ has a very short half-life and significant efforts have been made to determine the minimum effective dose and optimal route of administration of exogenous PGs (Colazo et al. 2002). In recent years, one of the most widely used PGF_2α_ analogs is cloprostenol (Abecia et al. 2011; Granados-Villarreal et al. 2017). Cloprostenol may be more advantageous because it can remain in circulation longer (Minela et al. 2021). Although increasing the dose of cloprostenol does not affect the half-life, half of the initial concentration is still present in plasma after this time (Whalen 2018). A positive relationship has been reported between the dose of PGF_2α_ and the percentage of ewes responding to treatment by showing estrus behavior (Pope and Cárdenas 2004; Fierro et al. 2013). In sheep, a low dose of PGF_2α_ (3 mg/60 kg) decreased P4 levels without luteolysis, while higher doses of PGF_2α_ (10 and 30 mg/60 kg) both decreased P4 levels and induced luteolysis (Juengel et al. 2000). In addition, repeating the dose of cloprostenol may have a significant effect on achieving complete luteolysis (Wiltbank et al. 2015). In a study conducted in cows, two doses of PGF_2α_ administered 24 h apart increased the proportion of cows with complete luteolysis before insemination compared to a single dose of PGF_2α_ (Brusveen et al. 2009). Studies on PGF_2α_ in sheep have generally involved single administration of different doses (Granados-Villarreal et al. 2017), and there are no studies investigating the luteolytic activity of reduced doses of cloprostenol at short intervals. In contrast, the use of two doses of cloprostenol at short intervals in cattle is quite limited (Brusveen et al. 2009). Using different reduced doses of cloprostenol can help identify differences in luteal sensitivity, which may be due to either the stage of the luteal phase or the initial progesterone (P4) concentrations (Granados-Villarreal et al. 2017). Some recent studies suggest that high P4 levels have a luteoprotective effect and protect the corpus luteum (CL) from luteolysis (Davis et al. 2010). High P4 levels at the time of PGF_2α_ treatment were associated with decreased sensitivity to reduced doses of cloprostenol, and the luteoprotective action of P4 does not depend on the luteal phase stage in ewes (Granados-Villarreal et al. 2017).

Since the luteolytic effect of PGF_2α_ can vary by the day of the estrous cycle and the dose, fixing the day of the cycle in dose studies can be beneficial. Presynchronization protocols can be used for this purpose. It was hypothesized that higher or split doses of cloprostenol, compared to lower doses, would exert a stronger luteolytic effect and consequently lead to improved reproductive performance in sheep. This study aimed to investigate the luteolytic effects of different and split doses of cloprostenol administered during the mid-luteal phase of the estrous cycle in ewes presynchronized with P4 and PGF_2α_, as well as their effects on the reproductive parameters and serum P4 levels in ewes.

## Materials and methods

### Animals

This study was conducted during the breeding season (July) at the Konya Bahri Dağdaş International Agricultural Research Institute. 147 Anatolian Merino sheep, aged 3–5 years, body condition score (BCS) 2.5–3.0 and dry, were used in the study. These ewes had no diseases and had given birth normally during the previous lambing season. The ewes were randomly allocated into three groups, ensuring equal distribution of age and body condition score (BCS) across the groups. This study was approved by the Animal Experiments Local Ethics Committee of Bahri Dagdas International Agricultural Research Institute (approval number: 31.01.2024-170). The study was conducted at an official research institute, where the conditions were highly suitable in terms of animal welfare.

### Protocols

**Fig. 1:**
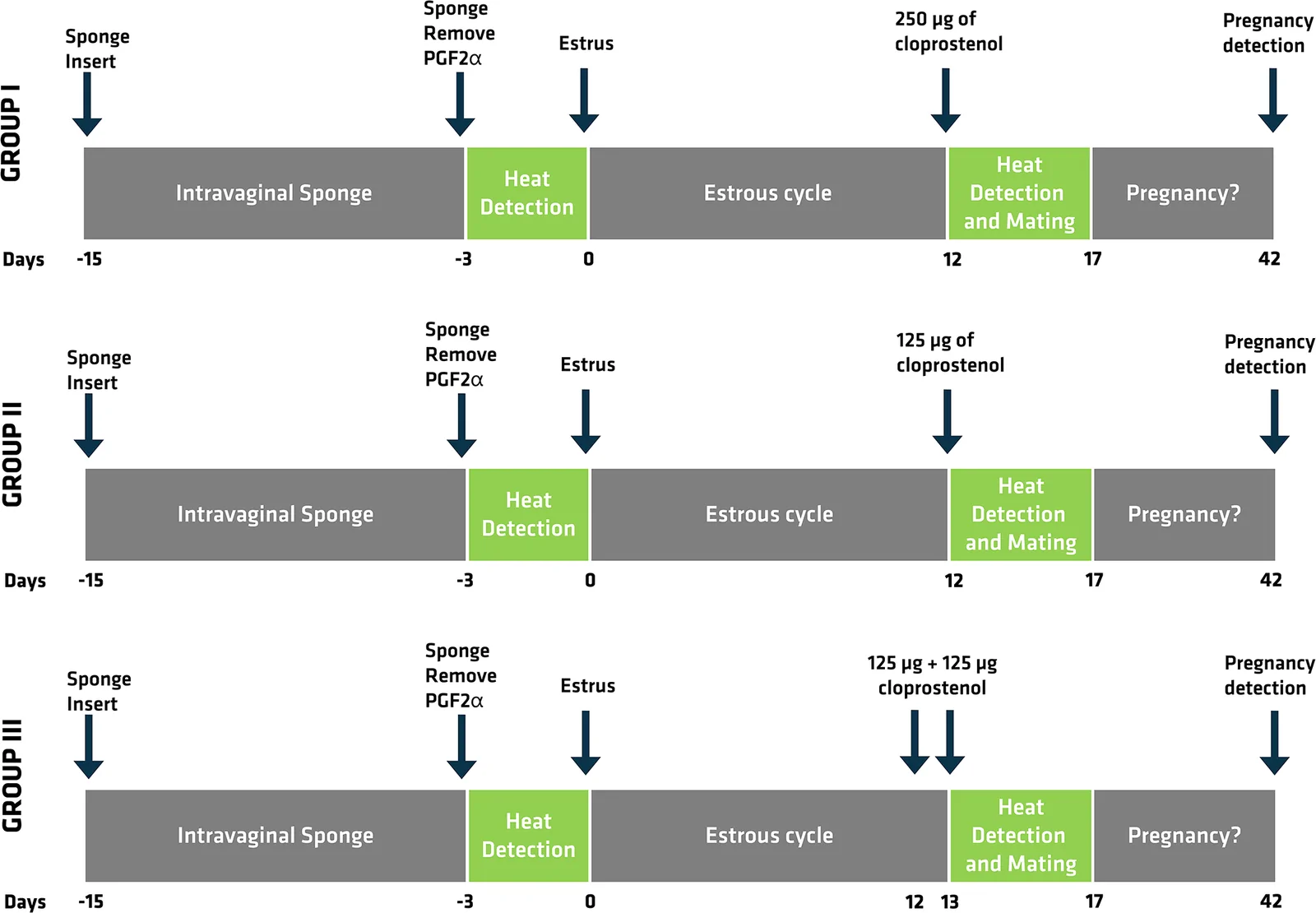
Days of synchronization protocol and practices in groups

The ewes were assigned to three groups. In real field conditions, presynchronization may not always be applied; however, in the present study it was implemented to ensure that the ewes were at the mid-luteal stage. Presynchronization protocol was applied to synchronize estrus in all groups, in which ewes were fitted with intravaginal sponges containing 60 mg of medroxyprogesterone acetate (Esponjavet, Hipra, Spain) for 12 days. After the sponges were removed, 250 µg of cloprostenol (Selexol, Pi Pharma, Ankara, Türkiye) was injected intramuscularly (IM), and teaser rams were used to detect estrus. The day on which estrus occurred was considered day 0. On day 12 of the estrous cycle, Group I (*n* = 47) received 250 µg of cloprostenol (1 dose) (Selexol, Pi Pharma, Ankara, Türkiye), Group II (*n* = 50) received 125 µg of cloprostenol (½ dose), and Group III (*n* = 50) received two doses of cloprostenol (2 dose) (125 µg on day 12 and 125 µg on day 13, with a 24-hour interval between doses (Fig. 1).

### Estrus detection and mating

Starting 16 h after the PGF₂α administration on day 12 of the estrous cycle, estrus detection was performed every 8 h using teaser rams. In accordance with the institute’s routine practice ewes detected in estrus were transferred to mating pens and hand-mated with fertile rams.

### Reproductive parameters

Estrus rate, pregnancy rate, twinning birth rate, and litter size were calculated as reproductive parameters (Kutlu et al. 2024) as follows;

$$\begin{aligned}&\mathrm{E}strus\:rate\cr &=\frac{the\:number\:of\:ewes\:showing\:estrus\:behaviours}{the\:number\:of\:ewes\:fitted\:with\:an\:intravaginal\:sponge}\:\cr &\times\:100\end{aligned}$$

$$\begin{aligned}&Pregnancy\:rate\cr &=\frac{the\:number\:of\:pregnant\:ewes}{the\:number\:of\:ewes\:fitted\:with\:an\:intravaginal\:sponge}\cr &\times\:100\end{aligned}$$

$$\begin{aligned}&Twinning\:birth\:rate\cr &=\frac{the\:number\:of\:multiple\:lambing\:ewes}{the\:number\:of\:pregnant\:ewes}\cr &\times\:100\end{aligned}$$

$$\begin{aligned}&Litter\:size\cr &=\frac{the\:number\:of\:total\:lambs}{the\:number\:of\:lambing\:ewes}\cr &\times\:100\end{aligned}$$

### P4 analysis

Blood samples were collected from the ewes in the groups on day 12 of the estrous cycle, starting immediately before the PGF_2α_ injection (0) and continuing at 12-hour intervals until estrus. Since estrus developed within 48 h in most ewes across all groups, the blood samples collected up to 48 h and on Day 5 of pregnancy were included in this study. Blood samples were collected from the jugular vein into vacutainers containing sodium EDTA and then centrifuged at 5,000 *g* for 10 min at 5 °C. Then, aliquots were stored in a − 20 °C freezer until analyzed. Plasma P4 levels were determined using the chemiluminescence enzyme immunoassay (CLIA) method with the commercial Alinity I Progesterone CMIA Kit (Abbott, USA). The intra- and inter-assay CV was 3.0% and 4.5% for progesterone while the assay sensitivity was 0.05 ng/mL.

### Pregnancy diagnosis

The pregnancy status of ewes was determined by the presence of an embryo with a heartbeat 25–30 days after mating, as determined by appropriate scientific methods. A transrectal linear probe operating at a frequency of 5–7.5 MHz connected to a portable B-mode ultrasonic device (DP 50 VET; Mindray Ltd., China) was used.

### Data analysis

The statistical analyses were performed using SPSS version 23. The sample size calculation was performed using G*Power software version 3.1.9.2. Assuming a medium effect size of 0.3 for detecting differences in P4 concentrations among groups, with an alpha level of 0.05 and a power of 0.80, the minimum required number of ewes was calculated as 111. Data normality was assessed using the Shapiro–Wilk test. The time between the PGF_2α_ injection and heat detection in each group was analyzed using survival analysis with the Kaplan–Meier method. Differences in the estrus rate, pregnancy rate, twinning birth rate, and litter size between groups were examined using the chi-squared test. At each sampling time, differences in P4 levels among the three groups were examined using one-way analysis of variance (ANOVA). Differences in P4 levels across different days within each group and the group*time interactions were examined using repeated measures two-way ANOVA.

The relationships between P4 levels were examined using Pearson correlation analysis. Statistical significance was set at *P* < 0.05. Results were evaluated as mean ± standard deviation (SD) for continuous variables and as percentage (%) for categorical variables.

## Results

### Luteolytic effect of different cloprostenol doses

**Fig. 2:**
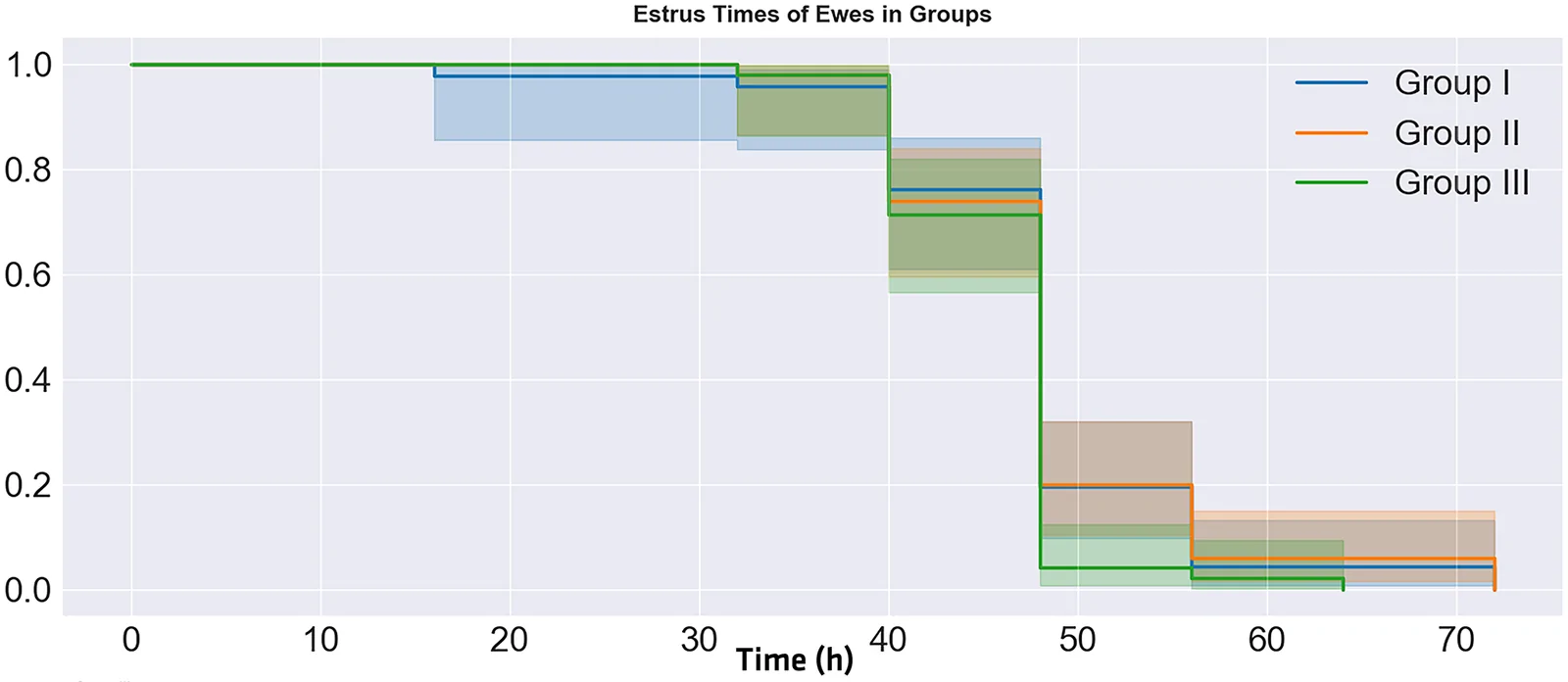
The Kaplan-Meier curve indicating the estrus time

The time (in hours) from cloprostenol injection to the observation of estrus signs in each group is shown in Table 1. A subsequent analysis revealed no statistically significant differences in the time to observation of estrus signs between the groups (*P* > 0.05). The percentages of sheep exhibiting estrus signs within the initial 48 h were as follows: 80.4% (37/46) in 1 dose, 80.0% (40/50) in ½ dose, and 95.9% (47/49) in 2 dose. The 2 dose rate was found to be statistically significant compared to the other groups (*P* < 0.05). Although estrus rates did not show a statistical difference among the groups up to 72 h, the group receiving split-dose cloprostenol (2 dose) exhibited a significantly higher rate by 48 h. This suggests that the split-dose cloprostenol regimen may accelerate the early estrus response. The Kaplan-Meier curve indicating the estrus time in the groups is shown in Fig. 2.

**Table 1:** Time of estrus after first Cloprostenol injection in groups

Groups	*n*	Estrus rate	Time (Hours)								
			16	24	32	40	48	56	64	72	Mean ± SD
Group I	47	98^a^ (46/47)	1	-	1	9	26	7	-	2	47.65^a^±8.75
Group II	50	100^a^(50/50)	-	-	1	12	27	7	-	3	48.32^a^±8.07
Group III	50	98^a^ (49/50)	-	-	1	13	33	1	1	-	46.04^a^±5.04

(*P* > 0.05). SD: Standard deviation

### Reproductive parameters

The reproductive parameters in each group obtained from the first service after PGF_2α_ treatment are presented in Table 2. The reproductive parameters, including estrus rate, twinning rate, and litter size, did not differ significantly between groups. However, the pregnancy rate did differ significantly between groups, with the pregnancy rate higher in 1 dose than in ½ dose (*P* < 0.05).

**Table 2:** Pregnancy, twinning rate and litter size of the groups

Groups	Data obtained from the first service		
	Pregnancy rate %	Twinning rate %	Litter size
Group I	85^**a**^ (39/46)	44^a^ (17/39)	1.44^a^ (56/39)
Group II	58^**b**^ (29/50)	41^a^ (12/29)	1.41^a^ (41/29)
Group III	71^**ab**^ (35/49)	37^a^ (13/35)	1.37^a^ (48/35)

There is a statistically significant difference (*P* < 0.05) between different letters in the same column(a, b)

### P4 concentrations

**Fig. 3:**
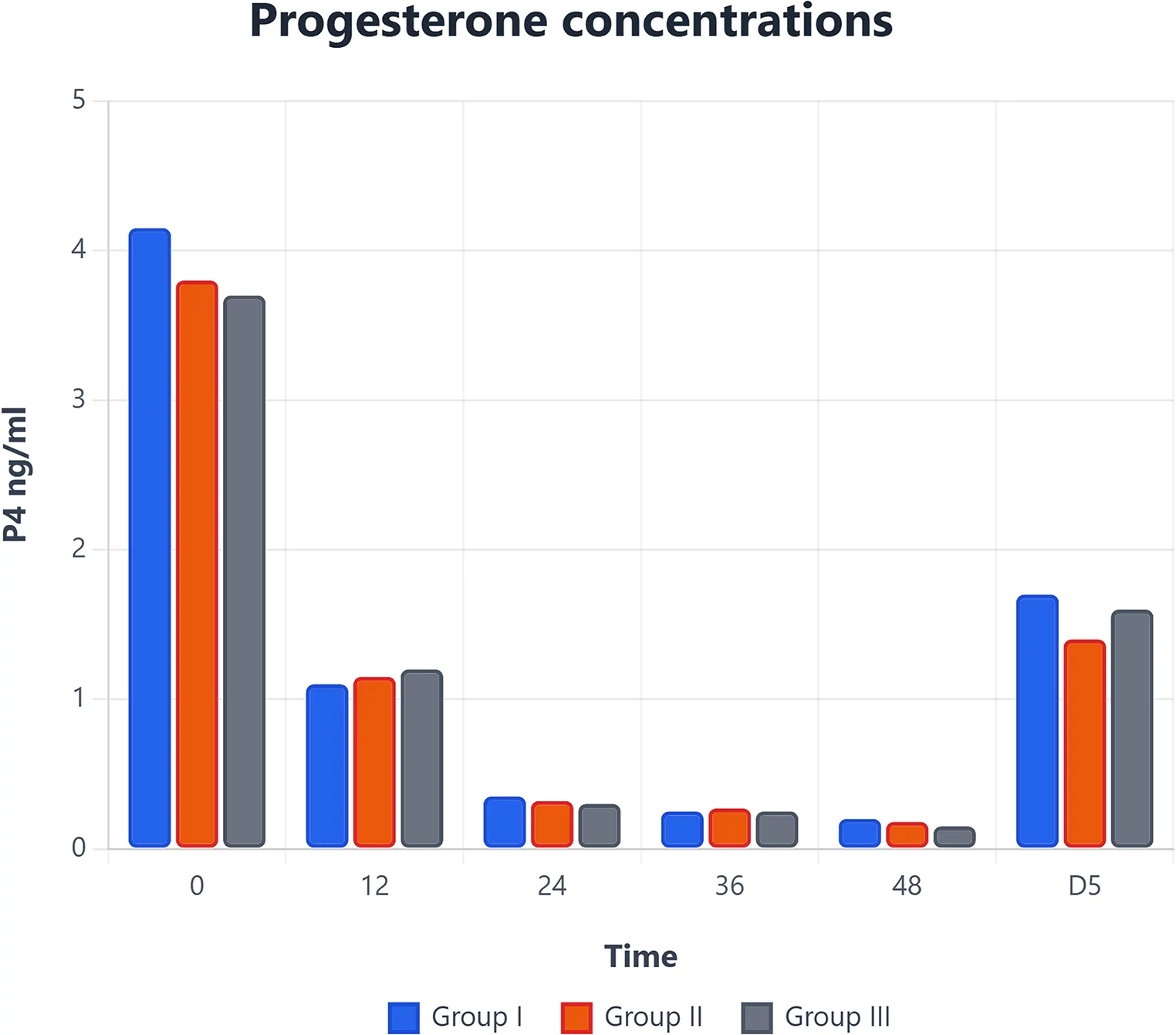
P4 concentrations during luteolysis (48 h) and on day 5 of gestation D5: day 5 of gestation

Plasma P4 concentrations in ewes were measured at 0, 12, 24, 36, and 48 h after the first cloprostenol injection and 5 days after mating. The concentrations were as follows: Group I: 4.19 ± 1.82, 1.08 ± 0.39, 0.33 ± 0.05, 0.26 ± 0.06, 0.22 ± 0.07, and 1.71 ± 0.44; Group II: 3.85 ± 1.24, 1.09 ± 0.40, 0.36 ± 0.12, 0.26 ± 0.06, 0.21 ± 0.06 and 1.39 ± 0.52; Group III: 3.73 ± 1.29, 1.15 ± 0.56, 0.34 ± 0.09, 0.26 ± 0.07, 0.19 ± 0.04 and 1.59 ± 0.54 respectively (Fig. 3). The group × time interaction on P4 levels was tested using two-way ANOVA, and no effect of the interaction was found. The correlation analysis of P4 levels on the indicated days, ignoring the groups, is presented in Table 3. Plasma levels of P4 were positively correlated between time 0 (day 12 of the estrous cycle) and the other time points except 48 h. Notably, plasma levels of P4 were moderately correlated between day 5 after mating and 0 and 12 h.

**Table 3:** Correlation analysis of P4 concentrations on different days

P4(0)		P4(12)	P4(24)	P4(36)	P4(48)	P4(D5)
	Correlation coefficient	0.705^**^	0.346^**^	0.412^**^	0.057	0.486^**^
	P	< 0.001	< 0.001	< 0.001	0.569	< 0.05
P4(12)	Correlation coefficient	1	0.454^**^	0.526^**^	-0.003	0.444^*^
	P		< 0.001	< 0.001	0.987	0.044
P4(24)	Correlation coefficient		1	0.305^*^	0.400^**^	0.247
	P			< 0.05	< 0.001	0.165
P4(36)	Correlation coefficient			1	0.278	0.137
	P				0.079	0.545
P4(48)	Correlation coefficient				1	0.156
	P					0.395

**. Correlation is significant at the 0.01 level. *. Correlation is significant at the 0.05 level

## Discussion

This study investigated the luteolytic efficacy of different cloprostenol doses and a split-dose regimen in ewes. In cattle, 500 µg cloprostenol is generally used as a luteolytic dose (Dudhatra et al. 2012). It has been reported that the PGF_2α_ dose used to induce luteolysis in ewes can be half of that used in cows (Ozbilek et al. 2022). A second cloprostenol dose may help to achieve better synchronization. CL sensitivity to PGF_2α_ gradually increases with luteal development, and this sensitivity may be further enhanced by repeated exposure to PGF_2α_ (Pate and Hughes 2023). In a previous study, estrus rates following administration of 25 mg PGF_2α_ on days 5, 8, and 11 of the estrous cycle were 85.4%, 84.0%, and 86.0%, respectively. Furthermore, in the same study, ewes administered PGF_2α_ on the day 5 of the estrous cycle showed a tendency towards a higher pregnancy rate at first insemination, a higher proportion of lambing ewes at first insemination, and a higher fertility rate at first insemination compared to other ewes. (Paul 2015). Additionally, a second PGF treatment in dairy cows during the Double Ovsynch protocol significantly reduced inadequate CL regression, resulting in an approximately 10% increase in pregnancy rates (Wiltbank et al. 2015). The high estrus rates in our study can be explained by cloprostenol being administered in the mid-luteal phase of the estrous cycle when the CL is sensitive to PGF_2α_. In addition, the estrus rate of up to 48 h in 2 dose may be due to the second dose preventing the delay in CL regression.

While the twinning rate and litter size did not differ significantly between groups, the pregnancy rate was significantly higher in 1 dose than in ½ dose (*P* < 0.05). In previous studies, cloprostenol was generally administered in two doses at different intervals or after intravaginal P4. One study involving ewes administered 25 mg of PGF_2α_ on days 5, 8, and 11 of the estrous cycle and reported that the pregnancy rates in the first service were 62.5%, 48.0%, and 44.0%, respectively (Paul 2015). Another study administered two different cloprostenol doses (125 and 250 µg) nine days apart in ewes and found no significant difference in estrus and conception rates (Kumar et al. 2018). In a study involving dairy cows, the cows were divided into three groups in the mid-cycle period, with 225 µg of d-cloprostenol administered as a single dose in Group I, two doses 12 h apart in Group II, and two doses 24 h apart in Group III, resulting in conception rates of 33.3%, 40.0%, and 42.2%, respectively and consequently, it has been reported that administering one or two doses of PGF₂α at 12- and 24-hour intervals during the mid-estrous cycle did not result in any difference in luteolysis, estrus, or pregnancy rates. (Neisari et al. 2022). The effect on gonadotropin regulation, PGF_2α_ has a local effect on the ovary (de Moraes et al. 2021). PGF_2α_ has been demonstrated to play a pivotal role in reducing follicular blood flow during the later stages of the ovulation process, particularly at the rupture point. Moreover, it has been hypothesized that prostaglandins may induce the activation of proteolytic enzymes, particularly collagenases, which have been implicated in follicular wall breakdown (Davies et al. 2006). One study involving ewes found an increase in ovulation rate due to the use of PGF_2α_ and reported that this increase could be due to the recruitment of ovulatory follicles from the previous and subsequent waves of PGF_2α_ application (Barrett et al. 2002).

In our study, although the increase in pregnancy rates observed in 1 dose may be attributed to a higher ovulation rate resulting from the cloprostenol dose, the lack of differences in twinning rate and litter size creates a discrepancy. Nevertheless, determining the number of ovulations or corpora lutea per ewe would provide a better insight into this matter. Although the decline in P4 levels in the half-dose group was similar to the other groups, it is also possible that luteolysis was insufficient or delayed. In our study, the lower pregnancy rates observed in the ½ dose cloprostenol group cannot be definitively explained. Future studies may investigate how dose variations influence hormonal fluctuations and the timing of ovulation.

Additionally, breed, season and pre-synchronization protocol implementation should be taken into consideration depending on the dose. Data of this study show that the doses and administration times of cloprostenol did not result in any variation in the rate of luteolysis across groups. A previous study that administered a standard single dose (500 µg), a single low dose (375 µg), and two low doses (375 µg and 250 µg 24 h apart) ) of cloprostenol in cows reported luteolysis rates of 60.0%, 66.7%, and 100%, respectively (Liu et al. 2017). Another study reported that ewes with low initial P4 levels exhibited increased susceptibility to cloprostenol, and luteolysis failed to occur in the presence of the luteoprotective effect of high initial P4 levels (Granados-Villarreal et al. 2017). In contrast, our study observed luteolysis in almost all ewes and no luteoprotective effect with high P4 levels. The reason for this is probably that all our ewes were in the middle of cycle and the CLs are in a fully responsive state. One study involving goats reported that different doses and administration methods of a PGF_2α_ analog (dinoprost) affected luteolysis rates. Its findings indicate that luteolysis was completed in all goats in the group given a single IM injection of dinoprost (2 mg). They also indicated that luteolysis rates were lower among goats given IM injections of dinoprost (1 and 2 mg) at 8-hour intervals than among those given a single IM injection of dinoprost (2 mg) (Akamatsu et al. 2023). The high rate of luteolysis in the groups in our study suggests that the doses of cloprostenol used in the mid-luteal phase (250 µg, 125 µg, and 125 + 125 µg) were sufficient to induce luteolysis in Anatolian Merino ewes.

In this study, an approximately 95% decrease in P4 levels was observed within 48 h after cloprostenol injection. CL size correlated positively with P4 levels after the induction of luteolysis with cloprostenol in heifers (Assey et al. 1993). It has been hypothesized that PGF_2α_ exerts its luteolytic effect on the CL, thereby inducing a substantial decline in blood flow. The first 2–3 h after the injection of PGF_2α_ are critical for the initiation of functional luteolysis, and it may cause a rapid increase in EDN1 release in the regressing CL during this period (Girsh et al. 1996; Ohtani et al. 1998). Our study also established positive correlations between P4 levels measured at 12-hour intervals within the first 36 h. However, it also determined that only the P4 levels at 24 h correlated with those at 48 h, during which estrus occurred intensely. Therefore, it can be concluded that the decrease in P4 levels up to 36 h is partially affected by the P4 level at baseline (0 h).

Apart from the main focus of our study, we also observed a correlation between P4 levels at 0 and 12 h and the P4 levels of pregnant ewes on day 5 after mating. Consequently, elevated P4 levels on day 12 of the estrous cycle (after the administration of cloprostenol) may precipitate a more pronounced increase in P4 levels during the early stages of pregnancy. It has been reported that P4 levels in the estrous cycle just before insemination and in the early luteal phase of pregnancy may affect pregnancy rates and embryonic losses (Lonergan 2011; Spencer 2013). One study reported that blood P4 levels obtained 12 days before the first insemination affected conception rates in Holstein and Jersey cattle (Fonseca et al. 1983). Many studies have indicated that P4 levels during PGF_2α_ induced luteolysis with the Ovsynch method in Holstein dairy cows substantially impact the likelihood of pregnancy (Strickland et al. 2010; Martins et al. 2011; Pursley and Martins 2011). Specifically, while early pregnancy P4 levels may be influenced by mid-luteal phase P4 concentrations prior to mating, additional comprehensive studies are required to clarify this relationship.

## Conclusions

As a result of this study,

- Different PGF_2α_ protocols did not alter the overall estrus rate;
- The pregnancy rate in the first service did not increase with the split dose compared to the full dose, but was adversely affected by the ½ dose application;
- All PGF_2α_ protocols effectively reduced progesterone, thus demonstrating that CLs in the middle of the cycle are highly sensitive to even lower doses of PGF_2α_ in sheep;
- Since the use of a single ½ dose of PGF_2α_ adversely affected first-service conception rates, avoiding the ½ dose regimen is recommended, whereas the split-dose protocol may be advised to achieve better synchronization of estrus timing.

## Supplementary Information

Below is the link to the electronic supplementary material.


Supplementary Material 1


## Data Availability

Data is provided within the manuscript or supplementary information files.
