# Supplementary material for: Evaluation of luteolytic activity and reproductive outcomes in ewes following single and split dose cloprostenol during the breeding season
Source: Trop Anim Health Prod. 2026 Feb 12;58(2):98. doi: 10.1007/s11250-026-04916-y (PMC12901238; doi:10.1007/s11250-026-04916-y)
Supplement: Supplementary file 1 — Supplementary Material 1 [file 11250_2026_4916_MOESM1_ESM.pdf]

CAMBRIDGE Proofreading & EDITING LLC

EDITING CERTIFICATE

This document certifies that the manuscript listed below was edited by Cambridge Proofreading LLC for English grammar, punctuation, spelling, and style. We endeavoured to ensure that the authors' intended meaning was not altered during the review. All amendments were tracked with Microsoft Word's 'Track Changes' feature, allowing the authors full control over the changes made. We bear no responsibility for revisions made to the document after our edit on the date listed below.

Document title:

Evaluation of Luteolytic Activity and Reproductive Outcomes Following Single and Split Dose Cloprostenol During the Breeding Season in Ewes

Document authors:

Neffel Kürşat Akbulut Yavuz Kal Mesut Kırbaş Fatih Aladağ Hasan Alkan

Date edited: 05/26/2025

Order number: 966-69-30

For queries or verification contact info@cambridgeproofreading.com

Cambridge Proofreading LLC is a registered company headquartered in Chicago, Illinois, with a global presence. Our Certificate of Good Standing can be found in the Illinois state business database.
